# Role of clinical judgment and tissue harmonic imaging ultrasonography in diagnosis of paediatric acute appendicitis

**DOI:** 10.1186/1749-7922-6-39

**Published:** 2011-11-16

**Authors:** Ossama Zakaria, Tamer A Sultan, Tarek H Khalil, Tamer Wahba

**Affiliations:** 1 Division of Pediatric Surgery, Departments of Surgery, Faculty of Medicine, Suez Canal University, Ismailia, Egypt; 2 Sporting Students' Health Insurance Hospital, Alexandria, Egypt; 3 Department of Surgery Faculty of Medicine, Meoufyia University, Egypt; 4 Department of Radiology, Faculty of Medicine, Suez Canal University, Ismailia, Egypt; 5 Department of Radiology, Students' Hospital Alexandria University, Egypt

**Keywords:** Acute appendicitis in Children, Tissue Harmonic Imaging (THI), Ultrasound scan, Appendicitis clinical score

## Abstract

**Background:**

Appendicitis is the most common surgical emergency in children; yet, diagnosis of equivocal presentations continues to challenge clinicians.

**Aim:**

The objective of this study was to investigate the hypothesis that the use of a modified clinical practice and harmonic ultrasonographic grading scores (MCPGS) may improve the accuracy in diagnosing acute appendicitis in the pediatric population.

**Patients & Methods:**

**Main outcome measures**

Sensitivity, specificity, and accuracy of the modified scoring system. Five hundred and thirty patients presented with suspected diagnosis of acute appendicitis during the period from December 2000 to December 2009 were enrolled in this study. Children's data that have already been published of those who presented with suspected diagnosis of acute appendicitis- to whom a special clinical practice grading scores (CPGS) incorporating clinical judgment and results of gray scale ultrasonography (US) was applied- were reviewed and compared to the data of 265 pediatric patients with equivocal diagnosis of acute appendicitis (AA), to whom a modified clinical practice grading scores (MCPGS) was applied. Statistical analyses were carried out using Z test for comparing 2 sample proportions and student's *t*-test to compare the quantitative data in both groups. Sensitivity and specificity for the 2 scoring systems were calculated using Epi-Info software.

**Results:**

The Number of appendectomies declined from 200 (75.5%) in our previous CPGS to 187 (70.6%) in the MCPGS (*P *> 0.05).

Specificity was significantly higher when applying MCPGS (90.7%) in this study compared to 70.47% in our previous work when CPGS was applied (*P *< 0.01). Furthermore, the positive predictive value (PPV) was significantly higher (95.72%) than in our previous study (82.88%), (*P *< 0.01). Overall agreement (accuracy) of MCPGS was 96.98%. Kappa = 0.929 (P < 0.001). Negative predictive power was 100%. And the Overall agreement (accuracy) was 96.98%.

**Conclusions:**

MCPGS tends to help in reduce the numbers of avoidable and unnecessary appendectomies in suspected cases of pediatric acute appendicitis that may help in saving hospital resources.

## Introduction

Certainty of clinical diagnosis is the most challenging task in clinical practice. It is relatively straight forward to look up the treatment once a correct diagnosis has been made. A single perfect diagnostic test for acute appendicitis does not exist [1-3].

Despite the number of algorithms and diagnostic tests available, about 20% of patients with appendicitis are misdiagnosed [3-9].

Presence of normal appendix ranges from 5-25% out of suspected cases of acute appendicitis [5,10-13]. Negative appendectomies were thought to be relatively harmless; nevertheless, they result in considerable unnecessary clinical and economic costs [14]. Even despite the uncertainty of diagnosis, appendicitis demands prompt treatment in order not to be neglected and misdiagnosed leading to progression of the disease with its associated morbidity and mortality that may include the risk of perforation which happens in approximately one third of the cases [5,15,16].

In an attempt to improve diagnosis, attention has turned to radiological imaging. The use of ultrasound scan (US) has been advocated as the readily available simple and fast imaging modality particularly in thin patients and children. A normal appendix is not frequently observed using gray-scale US [17,18]. On the other hand Harmonic imaging (HI) increases the contrast and spatial resolution resulting in artifact-free images, and has been shown to significantly improve abdominal ultrasonography. Only a handful of reports exist regarding its application in pediatric patients. Most of them do not encompass its use in acute appendicitis [19].

This work aimed to investigate and assess the hypothesis that the use of a modified clinical grading judgment and Tissue Harmonic Imaging ultrasonography (THI), as a modified score-aided diagnosis; MCPGS may improve the accuracy in the diagnosis of children with equivocal pictures of acute appendicitis and to compare these results with those of previously published data of CPGS [1,2].

## Patients and Methods

This two centers study was carried out during the period from December 2000 to December 2009. Data of pediatric patients with suspected acute appendicitis who underwent the clinical judgment and US score aided CGPS were reviewed; this data was published before [1].

This was a modification of previously published scoring methods [2,3] including certain subjective clinical parameters measured as 1 point such as fever of 38, anorexia and vomiting, tachycardia of more than 120 beats/minute. Abdominal pain parameters were also measured with special emphasis on guarding or rigidity, positive per-rectal examinations, however, positive rebound tenderness was given 3 points in this score method as well as other clinical, laboratory and harmonic US measurements (Table 1).

**Table 1:** Clinical Practice Guideline Scoring System (CPGS) [1]:

			1	0	Score
**Clinical data**	**General**	- Fever	Yes	No	
		- HR	> 120/min.	< 120/min.	
		- Vomiting	Yes	No	
		- Dehydration	Yes	No	
	**Abdominal**	***Abd. pain***			
		- Localized	Yes	No	
		- History of similar - attacks	No	Yes	
		- Character	Constant	Intermittent	
		- Severity	Intolerable	Tolerable	
		- Course	Progressive	Regressive	
		- Relief by antispasmodic	No	Yes	
		- Bowel Habit alteration	Yes	No	
		- Rebound tenderness	Yes (3)	No	
		- Guarding or rigidity	Yes	No	
		- +ve P.R. examination	Yes	No	
**Investigations**	**Laboratory**	- WBCs leukocytosis	Yes	No	
		- Urine analysis (Findings of UTI)	Yes	No	
	**Focused abdominal U.S**.	- Appendicitis or mass	Yes	No	
		- +ve findings in female Adnxae	No	Yes	
		- +ve findings in liver, Gall bladder, billiary passages	No	Yes	
		- +ve findings kidneys	No	Yes	
		- Free fluid	Yes	No	
**Total score**					

*Interpretation of results:*

**21 - 15 **= highly suggestive of appendicitis.

**14 - 8 **= Patient needs repeated evaluation for conclusive result.

**7 - 0 **= the diagnosis of acute appendicitis in not likely.

Two hundred sixty five (265) pediatric patients were the core of our current study. In those patients; the proposed usage of THI, clinical judgment and practice as a modified score aided system MCPGS was applied.

The MCPGS with twenty five variables including harmonic ultrasound (US) examination and a marker of inflammatory response was assessed in multivariate analysis using the finding of acute appendicitis at operation as the end point were enrolled in this study (Table 2). Exclusion criteria included those who were proved to have other causes of acute abdominal pain rather than acute appendicitis.

**Table 2:** Modified clinical practice and harmonic ultrasonographic grading score (MCPGS):

			1	0	Score
**Clinical data**	**General**	- Fever	Yes	No	
		- HR	> 120/min.	< 120/min.	
		- Vomiting	Yes	No	
	**Abdominal**	***Abd. Pain***			
		- Localized	Yes	No	
		- History of similar - attacks	No	Yes	
		- Character	Constant	Intermittent	
		- Severity	Intolerable	Tolerable	
		- Course	Progressive	Regressive	
		- Relief by antispasmodic	No	Yes	
		- Bowel Habit alteration	Yes	No	
		- Tenderness	Yes	No	
		- Guarding or rigidity	Yes	No	
		- +ve P.R. Associated intra- abdomin,. Disease	Yes No	No Yes	
**Investigations**	**Laboratory**	- High WBCs - Elevated CRP	Yes Yes	No No	
		- Urine analysis (Findings of UTI)	No	Yes	
	Tissue Harmonic U.S. RLQ	-Aperistaltic non- Compressible blind ended tubular structure	Yes	No	
		-Distinct thickened appendicial wall layers	Yes	No	
		- Outer diameter > 6 mm	Yes	No	
		-Target sign appearance	Yes	No	
		-Appendicolith(s)	Yes	No	
		-Periappendiceal fluid collection	Yes	No	
		- Echogenic Prominent pericecal fat Appendicolith	Yes	No	
		- +ve findings in female Adnxae	No	Yes	
**Total score**					

*Interpretation of results:*

**15 - 25 **= highly suggestive of appendicitis.

**8 - 14 **= Patient needs repeated evaluation for conclusive result.

**0 - 7 **= the diagnosis of acute appendicitis in not likely.

**Figure 1:**
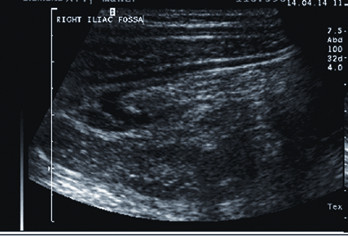
**Acute appendicitis by conventional US in a longitudinal scan using linear transducer with 7.5 MHz frequency showing a thick walled blind ended apristaltic non compressible inflamed appendix**..

**Figure 2:**
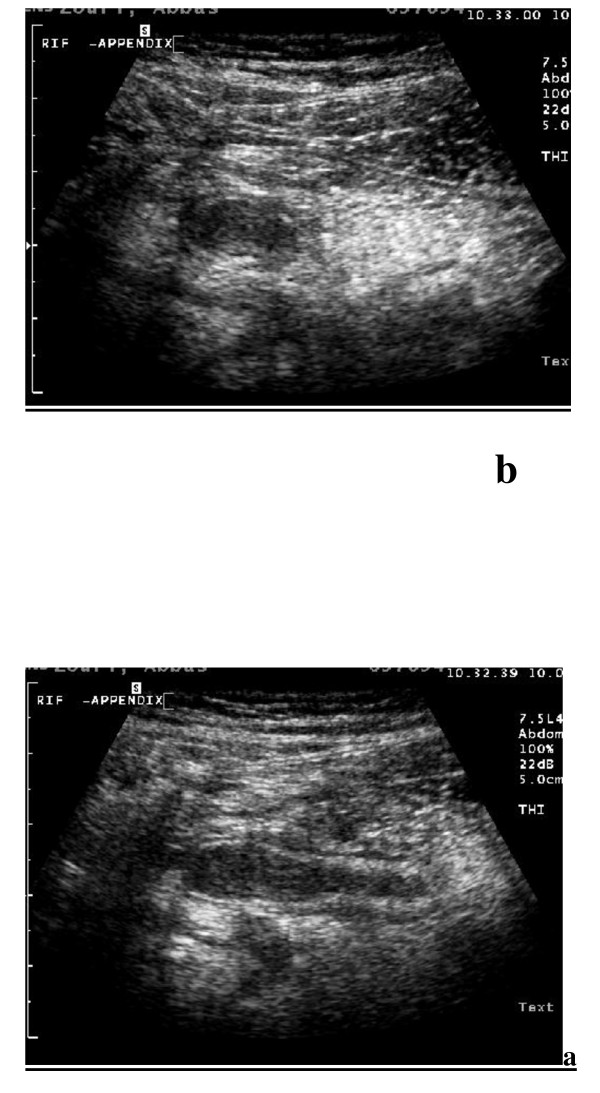
**Acute appendicitis by tissue harmonic imaging sonography (THI) using linear transducer with 7.5 MHz revealed: A**. Longitudinal scan showing aperistaltic non compressible blind ended tubular structure with distinct thickened wall layers and diameter > 6 mm. **B**. Transverse scan showing target sign appearance.

**Figure 3:**
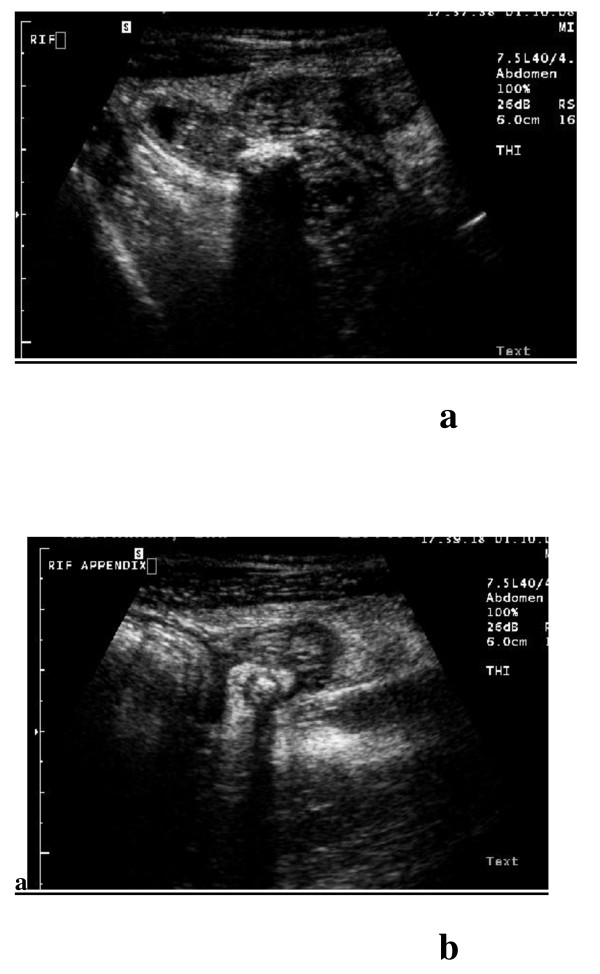
**Acute appendicitis by tissue harmonic imaging sonography (THI) using linear transducer with 7.5 MHz revealed: A**. Longitudinal scan showing a well defined adequately demarcated aperistaltic non compressible blind ended tubular structure with distinct thickened wall layers and diameter > 6 mm associated with intraluminal curvilinear calcification with posterior acoustic shadowing that reflects an appendicolith. **B**. Transverse scan showing target sign appearance with the appearance of the appendicolith with its characteristic posterior acoustic shadowing.

Ultrasonography was performed using linear and curved transducers with ultrasound frequencies ranged between 2.5 and 7.5 MHz, commercially available ultrasound systems (Siemens Sonoline Elegra, Germany). The examination was performed with both conventional and THI- US. Scanning parameters were optimized for each method, and all images were obtained with use of the same focal zone. A cine playback mode was used to obtain identical images in two standard planes, longitudinal and transverse scans. Images were obtained with the two methods in random sequence to facilitate their masking for the observers. Harmonic images were acquired at a transmitting frequency of 2.0 MHz and a receiving harmonic bandwidth of 4.0 MHz. Conventional US images were obtained at a frequency of 3.5 MHz, which is the commonly used frequency at abdominal imaging in adults. The harmonic and conventional US modes were switched by means of a toggle switch on the scanner control panel. In both the previous CPGS and the current MCPGS rationale of active watchful waiting in suspected appendicitis was a prudent and safe strategy with the use of at least one time repetition of conventional US or THI- US with no increase in the risk of perforation (Figures 1,2,3). All appendices were routinely sent for histopathological examination.

Collected data were statistically analyzed using *χ*^2 ^test. Continuous variables were analyzed using student's *t*-test. *P*≤0.5 were considered statistically significant. Sensitivity and specificity were calculated for the CPGS. Kappa test was used to verify the specificity. All calculations were performed using SAS version 8.2.

## Results

In the current studied group of patients; age and sex analysis shows that cases with and without appendectomy are similar and there is no aggregation of cases in a certain age group or in a certain sex (Table 3). In 187 patients (70.6%) appendectomy was performed, out of them 90 patients (48.1%) showed MCPGS between 15 and 22, those patients were kept with no oral feeding (NPO), intravenous fluid infusion (IV fluid) of appropriate type and amount according to patient's age before undergoing appendectomy. Only 8 out of the total appendectomies (4.3%) were normal at histopathological evaluation. The remaining 97 patients (36.6%) initially showed MCPGS of 8-14. On repeated evaluation every 2 hours for a maximum of 6 times and repetition of THI- US during the repeated evaluation for at least one time, their score progressed to 15 or more [61 patients (62.9%) with a MCPGS of 15-17, 11 patients (11.3%) with MCPGS of 18, and 25 patients (25.8%) with MCPGS of 19]. During the observation period, no antibiotics were given in order not to alter the clinical picture. However, antibiotics were started once the diagnosis was confirmed. No false negative cases were recorded when using MCPGS. (Tables 3, 4)

**Table 3:** Characteristics of studied children with clinically suspected appendicitis

Character	Number (%)
**Age (months)**	
Minimum-maximum (mean ± SD)	18-203 (140.63 ± 25.923)

**Gender**	
Male	159 (60.0%)
Female	106 (40.0%)

**Referring site**	
None (parent decision)	229 (86.4%)
Health establishment (Pediatrician)	36 (13.6%)

**Duration of symptoms before admission (hours)**	
Minimum-maximum (mean ± SD)	6-48 (23.15 ± 11.182)

MCPGS*	
Minimum-maximum (mean ± SD)	1-22 (11.54 ± 6.113)

**Final Outcome**	
No surgery	78 (29.4%)
Appendectomy with negative histopathology	8 (3.0%)
Appendectomy with positive histopathology	179 (67.6%)

MCPGS = Modified Clinical Practice Guideline Score

**Table 4:** Comparing characteristics of children with and without appendicitis

Character	With Appendicitis# (n = 179)	Without Appendicitis (n = 86)	Test *(P)*
**Age **(mean ± SD)	141.87 ± 23.584	138.06 ± 30.206	*t *= 1.12 (0.264)
**Gender**			X2 = 0.413 (0.520)
Male	105 (58.7)	54 (62.8)	
Female	74 (41.3)	32 (37.2)	
**Referring Agent**			X2 = 0.015 (0.903)
None	155 (86.6)	74 (86.0)	
Pediatrician	24 (13.4)	12 (14.0)	
**Duration **(mean ± SD)	22.54 ± 11.224	24.43 ± 11.051	Z = 1.497 (0.134)
**MCPGS **(mean ± SD)	14.82 ± 4.185	4.72 ± 3.120	12.393* (< 0.001)

* Significant, P < 0.05.

# include no surgery and surgery with negative histopathology

On the other hand, 78 children (29.4%) did not undergo appendectomy, 48 of them (61.5%) showed MCPGS of 8 or less at the initial examination, they were referred to the Pediatric Medical Care with no need for surgical interventions. Thirty patients (38.5%) showed MCPGS between 9 and 14 declining with repeated examinations until their score became definitely 8 or less, they were managed medically. (Tables 5, 6)

**Table 5:** Significant predictors of acute appendicitis using forward likelihood multiple logistic models

Predictor	β coefficient	Wald test	Exp B	95% Confidence Interval	
				
				LL	UL
**MCPGS**	0.795	50.851	2.214	1.780	2.755
**Duration**	-0.052	3.795	0.949	0.901	1.00
**Constant**	-5.187	25.711			

The model succeeded to correctly diagnose 95.5% of all cases, 97.2% of the positive cases, and 91.9% of the negative cases.

LL = Lower limit of the confidence interval of the odds ratio

UP = Upper limit of the confidence interval of the odds ratio (Exp B)

**Table 6:** Diagnostic screening criteria of MCPGS to detect children with acute appendicitis

MCPGS	Acute Appendicitis	Free	Total
**Positive score (8+)**	179 (100.0)	8 (9.3)	187 (70.6)
**Negative score (< 8)**	0 (0.0)	78 (90.7)	78 (29.4)
**Total**	179 (100.0)	86 (100.0)	265 (100.0)

Sensitivity = 100%

Specificity = 90.7%

Positive predictive power = 95.72%

Negative predictive power = 100%

Overall agreement (accuracy) = 96.98%

Kappa = 0.929 (P < 0.001)

**Figure 4:**
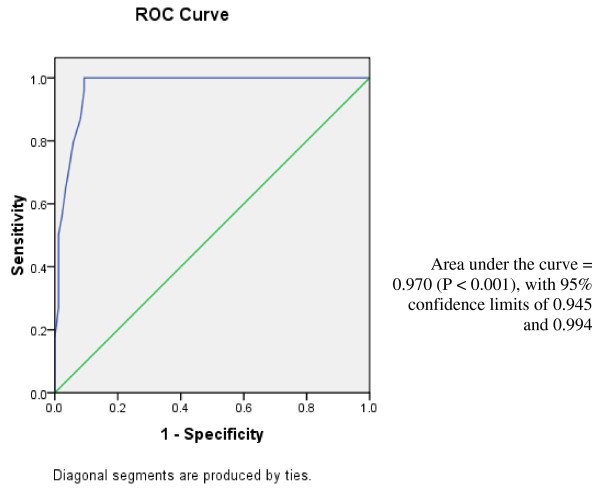
**Receiver operating Characteristics curve of MCPGS to detect children with acute appendicitis**. Area under the curve = 0.970 (P < 0.001), with 95% confidence limits of 0.945 and 0.994

Specificity of MCPGS was higher than that of CPGS, this may be attributed to the use of harmonic US in this modified scoring system that seems to be significantly superior to the conventional grey scale US 90.69% in group I (Table 5) compared to a specificity of 70.47% in group II (Z = 5.999, P < 0.01). Also the Positive Predictive Value for group II (95.72%) was significantly higher than that of group I (Z = 4.727, P < 0.01). Applying Kappa analysis revealed the Kappa Measure for over all agreement to be (96.98%). These results show the high specificity of our finding for the MCPGS. (Figure 4)

## Discussion

Acute appendicitis traditionally has been a clinical diagnosis and remains so to this day. The diagnosis can be difficult to make in many children who may present with typical symptoms or an equivocal physical examination [18].

In our current study, we evaluated the newly advocated modified clinical practice grading score (MCPGS); based on clinical judgment, laboratory investigations for inflammatory response and THI- US examination studies in association with the strategy of active watchful waiting performing repeated clinical examinations as well as at least one time repetition of THI before the decision-making process. It was highly accurate in the diagnosis of acute appendicitis in children. The specificity of the MCPGS was 90.69% compared to a specificity of 70.47% in the children to whom CPGS and active watchful waiting strategy was applied. In addition, we observed a statistically significant decrease in the negative appendectomy rate in MCPGS compared with those in CPGS.

Our study aimed at avoiding the selection bias mentioned before in similar scoring system [19]. Age and sex analysis shows that cases with and without appendectomy are similar and there is no aggregation of cases in a certain age group or in a certain sex. Therefore, the MCPGS can be used at any age and for any sex. Moreover, even those patients who were referred by pediatricians expected to be appendicitis were included as well as self referral that can be appendicitis or not. This illustrates that even if the cases are referred by pediatricians the score can still be used to differentiate cases.

The decrease in negative appendectomies occurred without a rise in the perforation rate. In fact, the perforation rate was lower under the MCPGS, although this change was not significant. Screening ultrasound scanning for pediatric appendicitis has suboptimal accuracy, particularly in obese children with a low likelihood of appendicitis who should not routinely undergo ultrasound scanning. However, when followed by a second ultrasound scanning or a clinical reassessment, it offers high diagnostic accuracy in lean children [20].

Targeted abdominal examination as well as THI constituted around 75% of our MCPGS scoring system with the aim of increasing its specificity without affecting the system sensitivity.

In our previously published data [1]; traditional clinical judgment and grey scale US score aided CPGS was performed, 200 patients (75.5%) underwent appendectomy, of them 35 appendices (17.5%) were normal at histopathological evaluation. The remaining 65 patients (24.5%) were discharged from the Pediatric Surgical Facility as not having appendicitis. Yet, out of those 65; 3 children (4.6%), (2 males and 1 female) were re-admitted. US was repeated suggesting acute appendicitis. They underwent appendectomy with positive pathological results. A total of 203 appendectomies (76.6%) were performed in this CPGS group.

Moreover, our current results showed the superiority of THI over conventional US for lesion visibility, with THI being preferred over conventional US for 65% of cases. The findings were clearer and better defined with THI which thereby improved the detection of subtle lesions. Tissue harmonic imaging theoretically improved signal-to-noise ratios by reducing noise from side lobe artifact in the near field and echo detection from multiple scattering events.

This reduced noise was most likely responsible for the superiority of THI over conventional US in the visualization of the findings and improved the confidence of diagnosis for most cases. THI was superior to conventional US in the visualization of lesions containing highly reflective tissues such as fat, calcium and air. It is therefore recommended to be used in obese patients. Better definition of the posterior acoustic shadows in calcifications and appendicolith(s) [21-28].

In our previous study the negative appendectomy rate was 17.5% compared to 4.3% in the current work. Contrary to our previous results [1] some published data expressed a negative appendectomy rate of 5.5% by applying somewhat similar scoring system [19]. The reason for such difference may be their use of computerized tomography scanning (CT) in their system. However, the difference in the negative appendectomy rate does not support the use of such an expensive sophisticated and hazardous radiological tool to children. CT scanning is not always available in all centers limiting its incorporation in clinical practice guideline scoring system. A recently published study of a practice guideline found that CT scan did not improve the accuracy of diagnosis in patients with suspected appendicitis [29]. Their guideline did not specifically address the appropriate use of CT scan.

Our MCPGS results, however, did show a great decline in the rate of negative appendectomies. This goes with data of some authors who showed that an imaging protocol using US followed by CT in their patients with equivocal presentations improved the accuracy of diagnosis of appendicitis [30].

We presented our results of MCPGS which evolved from this and other studies recommending ultrasound as the imaging modality of choice in most patients. In addition the recommendation of MCPGS was not limited to imaging alone. Most clinical practice scoring guidelines encourage, but do not require complaints with recommendations [31]. Measuring complaints can be challenging because scoring guidelines can include numerous recommendations and because patients, especially children do not always match preconceived scenarios [32]. Although many barriers limit physician acceptance of scoring guidelines [33], the compliance with our MCPGS is consistent with other developed practice scoring guidelines [2,3,6-9,34]. A considerable portion of the improvement seen in our study could be because of the utilization and accuracy of suitable imaging.

Practice scoring guidelines and clinical pathways have been implemented for many conditions [26], including acute appendicitis [16,30,35]. Analysis of such guidelines can focus on any combination of patient outcome, resource utilization or complaints with recommendation [16,34-38].

Although most appendicitis scoring guideline and pathways focus on decreasing postoperative treatment cost, a few concentrate diagnosis itself. One such pathway in a pediatric hospital achieved a significant reduction in the number of laboratory tests and X-rays without adversely affecting the incidence of negative appendectomies or perforation [34].

In our proposed MCPGS we included the minimum necessary laboratory investigations to measure the inflammatory response and time and effort saving tissue harmonic abdominal ultrasound scan in order to decrease the probabilities of misdiagnosing acute abdominal pain due to other reasons as acute appendicitis.

In our previous and current studies; all patients underwent the active watchful waiting strategy. This excludes that the decision-making process did result strictly from the MCPGS, and was not rather based on the repeated clinical re-evaluation that was adopted also on CPGS. This exactly shows that our proposed score is superior to the real life common clinical practice.

It may be concluded that the use of a modified clinical and THI ultrasonographic grading score (MCPGS) with the rationale of active watchful waiting in suspected appendicitis with at least one time repetition of THI-US was a prudent and safe strategy. It may improve the accuracy of diagnosing acute appendicitis in the pediatric population as it is superior to the real life common clinical practice.

It leads to fewer negative appendectomies compared with those children to whom it was not applied or other scoring systems were applied as the CPGS with the same strategy of active watchful waiting and repeated US, without a significant change in the perforation rate. Moreover, inpatient observation for serial examinations was reduced significantly. Our clinical practice grading scores can have considerable impact on the diagnosis of acute appendicitis in children. A larger cohort is necessary to validate our findings.

## Competing interests

The authors declare that they have no competing interests.

## Authors' contributions

OMZ has inspired the idea, collected the data and created the analysis and wrote most of the manuscript. TAS helped in collecting the data, analysis and writing of the manuscript. THK and TW have performed the sonography, collected the data and helped on manuscript writing. All authors read and approved the final manuscript.
